# Solubilization of Paclitaxel by Self-Assembled Amphiphilic Phospholipid-Mimetic Polymers with Varied Hydrophobicity

**DOI:** 10.3390/polym13162805

**Published:** 2021-08-20

**Authors:** Chie Kojima, Tomoka Hirose, Risa Katayama, Akikazu Matsumoto

**Affiliations:** Department of Applied Chemistry, Graduate School of Engineering, Osaka Prefecture University, 1-1, Gakuen-cho, Naka-ku, Sakai, Osaka 599-8531, Japan; sbb02100@edu.osakafu-u.ac.jp (T.H.); risa.katayama01@gmail.com (R.K.); matsumoto@chem.osakafu-u.ac.jp (A.M.)

**Keywords:** MPC polymer, hydrophobicity, polymer sequence, anticancer drug, solubilization, drug delivery

## Abstract

2-Methacryloyloxyethyl phosphorylcholine (MPC) polymers have been used as a coating agent on medical devices and as a carrier in drug delivery systems (DDSs). Paclitaxel (PTX) is a water-insoluble anticancer drug whose solubilizer is necessary for administration. Block and random copolymers composed of hydrophilic MPC and butyl methacrylate, named PMB, show different properties, depending on the polymer sequence and MPC content. In the present study, we used amphiphilic MPC polymers comprising hydrophobic dodecyl methacrylate (DMA). The self-assembling properties and PTX solubilization of random and block poly(MPC-co-DMA)s (rPMDs and bPMDs) with different compositions were examined and compared. rPMDs with high DMA content formed large and relatively loose self-assembled structures, which solubilized PTX. However, bPMDs formed small and compact self-assembled structures with poor PTX solubilization. PTX solubilized by PMB with small and loose self-assembled structures showed efficient drug action, similar to free PTX; however, rPMDs fell short of demonstrating PTX efficiency. Our results suggest that the self-assembling properties and the hydrophobicity of amphiphilic MPC polymers largely affect PTX solubilization as well as drug action, which is required to be controlled by the polymer sequence, as well as the structure and composition of the hydrophobic monomer for efficient DDS.

## 1. Introduction

2-Methacryloyloxyethyl phosphorylcholine (MPC) can mimic cell membranes and copolymerize with other vinyl monomers to produce various types of MPC polymers, which are applied to the surfaces of medical devices, such as cardiovascular devices, artificial joints, and contact lenses [1,2,3,4,5,6,7]. Some copolymers of MPC with hydrophobic comonomers were produced as a coating agent on medical devices. Random and block copolymers of MPC and butyl methacrylate (BMA) (rPMB and bPMB) and those of MPC and dodecyl methacrylate (DMA) (rPMD and bPMD) have been reported [3,4,8,9,10]. It is known that the self-assembling properties of MPC copolymers depend on the structure and composition of hydrophobic monomers and the sequence in polymers [11,12,13,14,15,16,17]. Dodecyl groups in PMD with high DMA contents interact with each other to form hydrophobic domains and micelles [11,12,13,14]. Block copolymers of PMB and PMD form micelles more efficiently than their random copolymers [13,16]. Self-assembled polymeric micelles have often been used for drug delivery systems (DDSs). In addition to MPC polymer-based micelles, polyethylene glycol (PEG) and zwitterionic polymers containing micelles have also been reported for the application to DDS [17,18,19,20,21,22].

**Figure 1:**
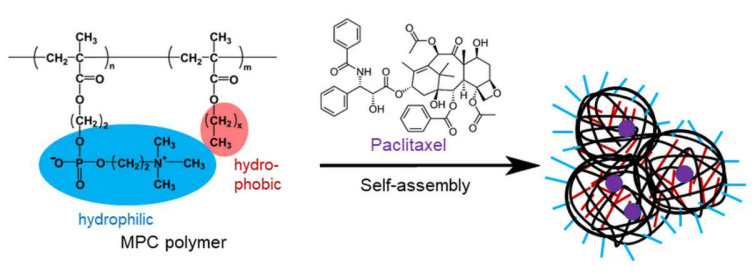
Chemical structure of poly(2-methacryloyloxyethyl phosphorylcholine (MPC)-co-n-butyl methacrylate) (PMB) (x = 3) and poly(MPC-co-dodecyl methacrylate) (PMD) (x = 11) and paclitaxel (PTX), and the self-assembly of MPC polymers. The compositions of each monomer (n and m) in PMB and PMD are shown in Table 1.

Paclitaxel (PTX) is a water-insoluble anticancer drug, and its solubilization is indispensable for administration. Although ethanol and polyethoxylated castor oil are used as solvents for PTX, they cause side effects, such as peripheral neurotoxicity, arthralgia, allergy, and hypersensitivity reactions. Additionally, PTX is not applicable to patients with hypersensitivity to alcohol [23,24]. Thus, solubilizers and/or carriers for PTX have been extensively investigated. Albumin, liposomes, and polymeric micelles have been studied for the solubilization and delivery of PTX. Some of them, for example, Abraxane^®^, Lipusu^®^, and Genexol-PM^®^, have been clinically approved [25]. Konno et al. reported that rPMBs played a role as a solubilizer of PTX, whose solubility was enhanced more than ten thousand times [26]. PTX solubilized by rPMB showed in vitro and in vivo antitumor effects [27]. bPMB and rPMB, which exhibited different self-assembling properties, showed different PTX solubilization effects [15]. Our previous report suggests that the self-assembling properties of PMDs are largely affected by the compositions of monomers and sequence in polymers [13]. However, PTX solubility using various PMDs has not been investigated to date. In the present study, we examined the relationship between the self-assembling properties of rPMDs and bPMDs with different compositions and PTX solubility. The drug action of PTX solubilized by PMD was examined and compared to free PTX and PTX solubilized by PMB (Figure 1).

## 2. Materials and Methods

### 2.1. Materials

PMBs and PMDs used in this study are listed in Table 1. PMBs and PMDs with high molecular weight were used in this study, which were the same polymers used in our previous reports [11,12]. The molecular weight of these polymers was estimated by static light scattering (SLS) using DLS-7000 (Otsuka Electronics Co., Ltd., Osaka, Japan) at 25 °C, whose results were presented in our previous report [13].

rPMDs were synthesized in our previous report [13]. Briefly, MPC, DMA, and 2,2’-azobis(isobutyronitrile) (AIBN) were dissolved at different monomer ratios in the mixture of ethanol/THF, in which the total monomer concentration was 1.0 mol/l. After the cycle of freezing–degassing–thawing and the bubbling of nitrogen gas, the polymerization was performed at 60 °C for 24 h. rPMDs were purified by the re-precipitation from the mixture of diethyl ether/chloroform, followed by vacuum drying. bPMDs were also synthesized in our previous report via reversible addition−fragmentation chain transfer (RAFT) polymerization [13]. Briefly, MPC, AIBN, and 4-cyano-4-[[(dodecylthio)carbonothioyl]thio]pentanoic acid] were dissolved in ethanol. After the cycle of freezing–degassing–thawing and the bubbling of nitrogen gas, the polymerization was performed at 68 °C for 24 h to obtain the PMPC-RAFT. After the purification of PMPC-RAFT, PMPC-RAFT, DMA, and AIBN were dissolved in a mixture of ethanol/chloroform at a volume ratio of 3/7. After the cycle of freezing–degassing–thawing and the bubbling of nitrogen gas, the polymerization was performed at 60 °C for 6 h. bPMDs were also purified by the re-precipitation from the mixture of diethyl ether/chloroform, followed by vacuum drying. rPMDs and bPMDs were characterized by ^1^H NMR analysis using ECS-400 and ECX-400 spectrometers (JEOL Ltd., Tokyo, Japan) and gel permeation chromatography (GPC), whose results were presented in our previous report [13].

### 2.2. Dynamic Light Scattering Analysis

The average hydrodynamic diameters were measured by dynamic light scattering (DLS) using ELSZ-DN2 (Otsuka Electronics Co., Ltd.). The MPC polymers dissolved in ethanol were diluted by water to prepare 1 mg/mL of MPC polymer solutions in an ethanol/water mixture of 1/29 (*v*/*v*). The diameter was estimated from the cumulant method.

### 2.3. Fluorescence Spectrometry

Sodium 1-anilino-8-naphthalene sulfonate (ANS) was dissolved in water to prepare a 1 mM ANS aqueous solution. Then, 20 mg/mL of various MPC polymer ethanolic solutions were prepared and diluted in water to prepare various MPC polymer solutions with concentrations ranging from 10^−5^ to 1 mg/mL. Further, 30 μL of the ANS solution was added to 2.97 mL of various MPC polymer solutions at different concentrations. The fluorescence spectra were measured upon excitation at 380 nm and recorded with an FP-6200ST fluorescence spectroscope (Jasco Inc., Tokyo, Japan). The maximum ratio of ethanol/water was 1/9 (*v*/*v*), at which the ANS fluorescence spectrum was similar to that in water. The maximum wavelengths of the ethanol/water and water were 511 nm and 518 nm, respectively. Pyrene was dissolved in ethanol to prepare a 50 μM pyrene solution, which was added to the various MPC polymer solutions at different concentrations. The fluorescence spectra from 350 nm to 460 nm were measured and recorded upon excitation at 336 nm. The ratio of the fluorescence intensity at 374 nm (I_1_) to that at 385 nm (I_3_) was calculated [15].

### 2.4. PTX Solubilization

Here, 5 μL of PTX solution (10 mg/mL in ethanol) and 15 μL of the various MPC polymer solutions (20 mg/mL in ethanol) were mixed. Then, 10 μL of ethanol and 270 μL of water were added to the ethanol mixture (0.1 mg/mL in PTX and 1 mg/mL in MPC polymers at 1/9 (*v*/*v*) of ethanol and water) in this order, followed by vortexing. The insoluble PTX was removed by filtration (φ 0.45 μm). PTX solubility was estimated from the standard curve of the PTX ethanoic solution by high-performance liquid chromatography (HPLC). The HPLC system was equipped with a column, Cosmosil 5C18-MS-II (Nacalai Tesque, Inc., Kyoto, Japan), and a UV detector (210 nm, UV-2075Plus; Jasco Inc.). Sample solutions (25 μl) were injected and eluted with a mixture of methanol and water at 70/30 (*v*/*v*) at 1.0 mL/min by PU-2089Plus (Jasco Inc.). PTX solubility in water was below the detection limit without any MPC polymers. Encapsulation efficiency (%) was calculated from the following equation:

Encapsulation efficiency (%) = [solubilized PTX (mg)]/[PTX in feed (mg)] × 100

The same experiments were performed by increasing the PTX concentration in the mixture of MPC polymers (1 mg/mL). For the cytotoxicity assay, PTX (0.2 mg/mL) and MPC polymers (1 mg/mL) were mixed in a 1/9 (*v*/*v*) ethanol–water mixture. The PTX concentration was confirmed by HPLC analysis prior to the cell assay.

### 2.5. Cytotoxicity

MDA-MB-231 cells were cultured using Dulbecco’s modified Eagle’s medium containing 10% fetal bovine serum [28]. For this, 6 × 10^3^ cells were seeded per well in a 96-well plate and cultured at 37 °C for 24 h. The PTX solutions containing PMB and rPMD34 were added to the culture medium to produce 1 and 1000 nM PTX solutions, which were added to the cells. After incubation for 48 h, the cells were washed with phosphate buffer saline, and 3-(4,5-dimethylthiazol-2-yl)-2,5-diphenyl tetrazolium bromide (MTT) assay was performed according to our previous report to evaluate cytotoxicity [28].

## 3. Results

### 3.1. Self-Assembly of rPMDs and bPMDs

rPMDs and bPMDs with different compositions were obtained in our previous report [13]. The polymer sequences, compositions, molecular weights, and diameters of PMDs are listed in Table 1. The self-assembling properties of these polymers were examined and compared. Because PMB is more hydrophilic than PMD, the high molecular weight of PMB is necessary for the applications [2,4,13]. PMD with a similar composition to PMB and high molecular weight was also used. The hydrodynamic diameters of these polymers were measured by DLS using 1 mg/mL of the polymer solution. The PMD with the low MPC contents formed submicron-sized aggregates. The aggregate size increased with the decreasing MPC content. In addition, rPMD66 was larger than bPMD65, suggesting that compact self-assembled structures were formed in block copolymers because of the dense packing of the hydrophobic units. The light scattering intensities of some polymers, such as PMB and PMDs with high MPC contents, were too small to obtain the diameter, suggesting that their size was <100 nm. The small diameter may be difficult to detect because the refractive indices of MPC and water are similar.

**Figure 2:**
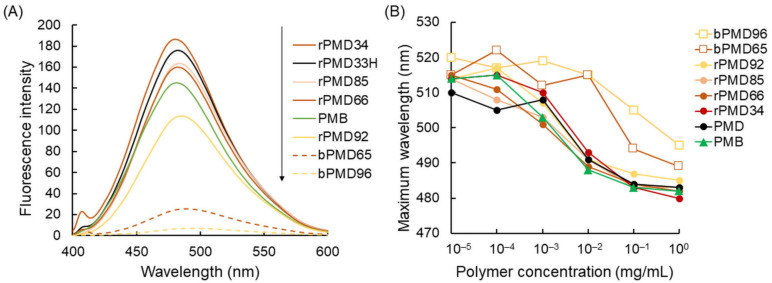
Hydrophobicity estimated using sodium 1-anilino-8-naphthalene sulfonate (ANS). (**A**) Fluorescence spectra of ANS in MPC solutions at 1 mg/ml. (**B**) Maximum wavelength of ANS at various MPC polymer concentrations.

The self-assembling property was also analyzed by ANS, which is a fluorescence dye whose spectrum changes with the hydrophobicity of the environment [15]. The spectrum is enlarged and shifted to a short wavelength under the hydrophobic environment. The fluorescence spectra of ANS in 1 mg/mL of various MPC solutions are shown in Figure 2A. Larger ANS spectra with shorter wavelengths were observed in rPMD with the low MPC content (the high DMA content), suggesting that the DMA content affected the hydrophobicity of the self-assembled structure. ANS spectra in rPMDs were similar and larger than those in PMB; on the contrary, those in bPMDs were much smaller, suggesting that bPMDs unexpectedly formed less hydrophobic assembly than rPMDs. This may be because of the smaller structures formed in bPMDs than in rPMDs. It is also likely that hydrophilic ANS could not detect dodecyl-assembled hydrophobic domains because ANS is soluble in water, alcohol, and acetone, but not in ethyl acetate and hexane. The ANS spectra were measured in different concentrations of various MPC polymers, and the maximum wavelengths in the fluorescence spectra were plotted against the MPC concentrations (Figure 2B). The maximum wavelength was 511 nm in the absence of any MPC polymers. The maximum wavelength tended to be shifted above 10^−3^ mg/mL, suggesting that all MPC polymers formed self-assembled structures. Although the diameters of some MPC polymers, such as PMB, were not detected by DLS analysis, smaller and/or unstable self-assembled structures were possibly detected by hydrophilic ANS.

**Figure 3:**
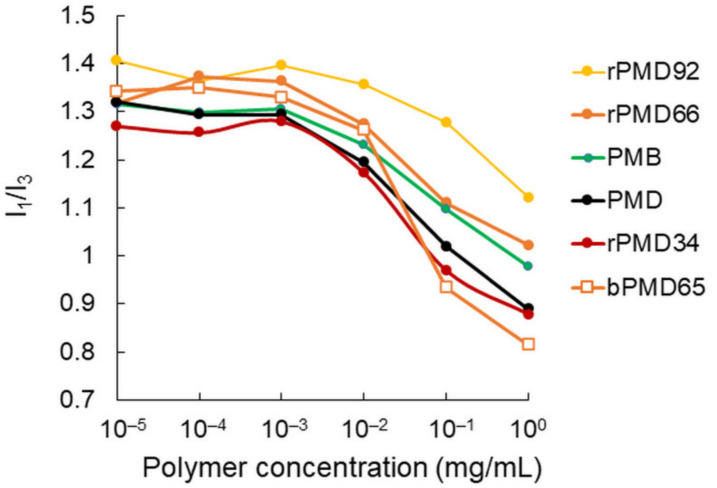
Hydrophobicity estimated using pyrene. The I_1_/I_3_ values in pyrene fluorescence spectra were plotted against the MPC polymer concentration.

Pyrene is a hydrophobic fluorescence dye that can also be used for evaluating hydrophobicity. It is known that the ratio of the first peak intensity (I_1_) to the third one (I_3_) in the fluorescence spectrum is correlated to hydrophobicity. The I_1_/I_3_ ratio was estimated from the pyrene fluorescence spectra in the presence of various MPC polymer solutions at different concentrations (Figure 3). The I_1_/I_3_ values tended to decrease above 10^-3^ mg/ml. For comparison among various MPC polymers at 1 mg/ml, rPMDs with lower MPC contents showed smaller I_1_/I_3_ values, indicating that the hydrophobic DMA content affected hydrophobicity, as confirmed by the ANS experiment. However, the I_1_/I_3_ value of bPMD66 was smaller than that of rPMD65, suggesting that bPMD formed more hydrophobic assembly than rPMD, contrary to the results of the ANS experiment. Our results indicate that the self-assembling properties of these MPC polymers were different, depending on the polymer sequence as well as the hydrophobic structure and content. PMB possibly formed small and loose aggregates, as previously reported [15]. rPMDs with high DMA content formed large and relatively loose aggregates, but bPMDs formed small and compact aggregates. This tendency was also observed in bPMBs and rPMBs [15].

### 3.2. PTX Solubility of rPMDs and bPMDs

**Figure 4:**
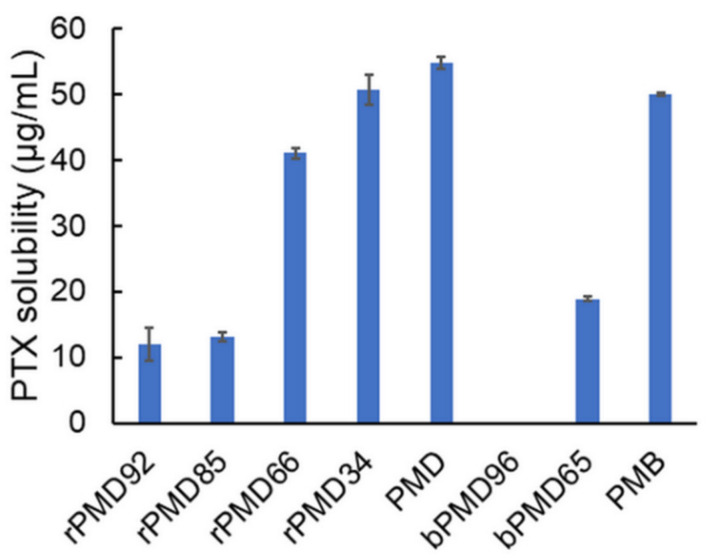
PTX solubility of MPC polymers at 1 mg/mL.

The solubilization of PTX was attempted using various MPC polymers. MPC polymers dissolved in ethanol were mixed with the PTX ethanoic solution at a ratio of 1/10 (*w*/*w*) PTX/PMDs. Then, water was added to produce a 1/9 (*v*/*v*) ethanol and water mixture. The final concentrations of PTX and PMDs were 0.1 and 1 mg/mL, respectively. After the insoluble PTX was removed by filtration, PTX solubility was estimated by HPLC analysis. PTX could be solubilized by various MPC polymers, except bPMD96 (Figure 4). The PTX solubility increased with the increasing DMA content, because the hydrophobicity in PMDs increased with the increasing DMA content. rPMDs could solubilize PTX more efficiently than bPMDs. PTX is easily dissolved in ethanol, but not in hexane. The PTX solubility is similar to that of ANS. Thus, the results of PTX solubilization were similar to those obtained from the ANS experiment. The PTX solubility in rPMD34 and PMD was the highest among PMDs, which was similar to that in PMB. When MPC polymer solutions in a 1/9 (*v*/*v*) ethanol and water mixture were prepared prior to mixing with PTX, PTX was not solubilized at all. Thus, the mixing of PTX and MPC polymers in ethanol is indispensable for solubilization of PTX.

**Figure 5:**
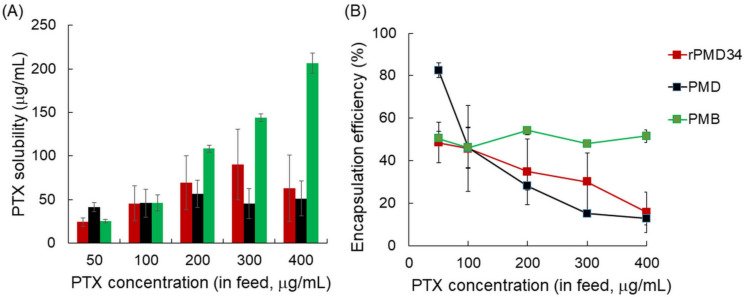
(**A**) PTX solubility and (**B**) PTX encapsulation efficiency (%) of MPC polymers mixed with different concentrations of PTX solutions.

Next, the mixing ratios of PTX and MPC polymers were varied to improve PTX solubility (Figure 5). PTX solubility increased gradually by increasing the PMB concentration, whereas the PTX encapsulation efficiency (%) was unchanged at around 50%. Meanwhile, PTX solubility did not increase with the increase in the rPMD concentrations. The PTX encapsulation efficiency (%) decreased as the PMD concentration increased. This suggests that the PTX solubilization mechanisms of PMB and PMD differ, which is described in the discussion.

### 3.3. Cytotoxicity of PTX Solubilized by Different MPC Polymers

**Figure 6:**
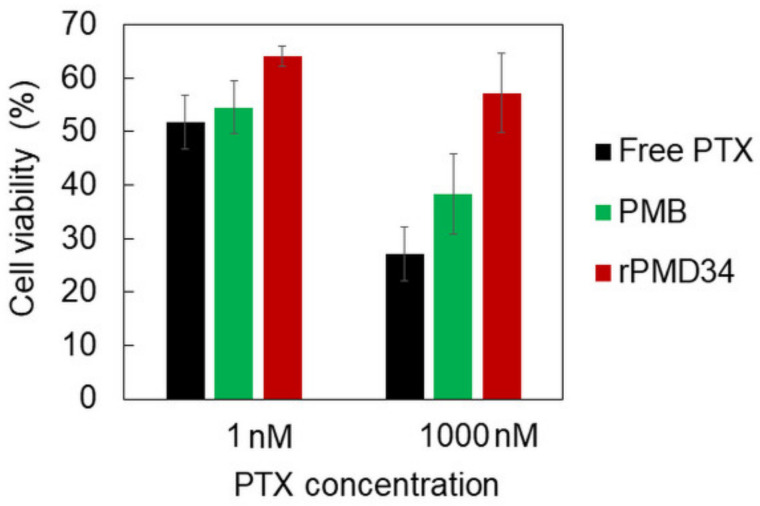
Cytotoxicity of free PTX and PTX solubilized by PMB and rPMD34 (n = 6).

The cytotoxicity of PTX solubilized by PMB and rPMD34 was compared against human breast cancer cells, MDA-MB-231 cells. Figure 6 shows the cell viability at 1 nM (~1 ng/mL) and 1000 nM (~1 μg/mL) PTX. Free PTX dissolved in ethanol was also examined as a control. PTX solubilized by PMB showed cell viability similar to that of free PTX, but PTX solubilized by PMD did not show efficient drug action even at high concentrations, likely owing to the different PTX solubilization mechanisms of these polymers. The MPC polymer concentration in this experiment was ~10^−5^ mg/mL for 1 nM PTX and ~10^−2^ mg/mL for 1000 nM PTX. Because the self-assembly of these MPC polymers was observed above 10^−3^ mg/mL, our results suggest that different self-assembling properties affect the cytotoxicity of PTX solubilized by these MPC polymers.

## 4. Discussion

**Figure 7:**
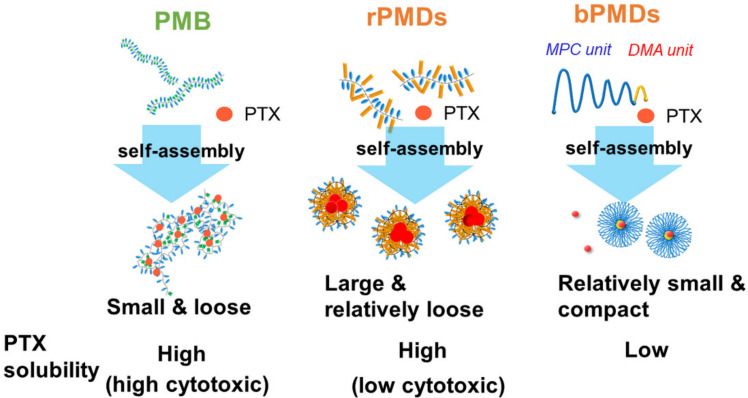
Schematic of the self-assembly and PTX solubility of various amphiphilic MPC polymers.

In the present study, the self-assembling properties were examined using two solvatochromic fluorescence dyes, ANS and pyrene, and DLS analysis. Amphiphilic MPC polymers were self-assembled above 10^−3^ mg/mL. The sizes and the hydrophobicities of self-assembled structures were different among the polymers. MPC polymers with high DMA content (low MPC content) efficiently interacted with each other, indicating that the DMA content was one of the main factors for self-assembly. The hydrophobicity in rPMDs and bPMDs was differently evaluated by ANS and pyrene, because ANS was less hydrophobic than pyrene. Altogether, rPMDs formed larger and relatively looser self-assembled structures, whereas bPMDs showed small and compact structures. In addition, our results suggested that PMB formed small and loose self-assembled structures, as reported in a previous report [15]. It is likely that the different self-assembling properties of PMDs affected PTX solubility. rPMDs with high DMA content efficiently solubilized PTX at a similar level to PMB, but bPMDs did not. PTX has some polar groups, which may not be highly associated with the hydrophobic domains made from dodecyl groups. Our results suggest the different PTX solubilization mechanisms of various amphiphilic MPC polymers, as shown in Figure 7. PMB interacted with PTX in weak affinity because of the loose self-assembled structures. On the contrary, the self-assembled structures of rPMDs were large and relatively stable; thus, PTX interacted more tightly with rPMDs than PMB. bPMDs formed more compact self-assembled structures, which did not solubilize PTX efficiently.

Similar phenomena were reported for rPMBs and bPMBs. More PTX was solubilized by rPMB with loose self-assembled structures than by bPMB with stable self-assembled structures [15]. It was also reported that PTX solubilized by rPMB was transferred to a serum protein because of the loose and flexible complexes of rPMB and PTX [15]. Thus, the drug action may be involved in the self-assembled structures. PTX loaded in PMB was released from the polymer and interact with serum proteins, similar to free PTX. However, PTX loaded in PMD may be stably encapsulated in the self-assembled structures. Because MPC polymers do not interact with cells and proteins, PTX in the PMD complex cannot be efficiently internalized into tumor cells. Our results suggest that the dodecyl group in PMDs is too hydrophobic for the solubility and release of PTX. However, the presence of the stable complex may be useful for the delivery to a target site. It was reported that ligand conjugation to MPC polymers could induce the uptake of target cells. Moreover, the addition of stimuli-sensitive units and biodegradable units to MPC polymers improves the drug release from the self-assembled structure [17]. Further improvement, for example, the addition of ligands and stimuli-sensitive and/or degradable units, is necessary for efficient PTX delivery using PMDs.

## 5. Conclusions

In the present study, we examined the self-assembling properties and PTX solubility of various types of amphiphilic MPC polymers. rPMDs with higher DMA content could solubilize PTX, which were self-assembled to form larger particles. The PTX solubility of rPMDs with large and relatively loose self-assembled structures was similar to that of PMB with small and unstable self-assembled structures, but higher than that of bPMDs with small and compact self-assembled structures. The cytotoxicity of PTX solubilized by rPMD was lower than that solubilized by PMB, which may result from the different self-assembled structures. Our results indicate that the self-assembling properties of amphiphilic MPC polymers highly affect PTX solubility, which can be controlled by the polymer sequence and the structure and composition of the hydrophobic unit. It is also suggested that the matching of the solubilizer and the drug in hydrophobicity is important. Our results provide an important insight to design a suitable self-assembled structure specific to a target drug for developing efficient DDSs.

## Figures and Tables

**Table 1:** PMB and PMDs used in this study.

Polymer	Sequence	Composition (mol%)		M_n_ ×10^4^ (Da)	M_w_ ×10^4^ (Da)	M_w_/M_n_	Diameter ^e^ (nm)
		MPC	BMA/DMA				
PMB	random	30	70	NA	46 *^b^*	NA	ND
PMD	random	33	66	NA	42 *^b^*	NA	740 ± 170
rPMD34	random	34	64	- *^a^*	- *^a^*	NA	700 ± 80
rPMD66	random	66	34	4.8 *^c^*	8.1	1.7	240 ± 20
rPMD85	random	85	15	4.8 *^c^*	13.3	2.8	ND
rPMD92	random	92	8	4.9 *^c^*	11.8	2.4	ND
bPMD65	block	65	35	5.1 *^d^*	NA	NA	130 ± 20
bPMD96	block	96	4	3.1 *^d^*	NA	NA	ND

The composition, molecular weight, and polydispersity were reported in our previous study [13]. ^a^ Insoluble. ^b^ Estimated from static light scattering (SLS) analysis. ^c^ Estimated from gel permeation chromatography (GPC) analysis. ^d^ Calculated from the theoretical degree of polymerization and the composition of the block polymers. ^e^ Measured by dynamic light scattering (DLS) analysis. NA: not applicable. ND: not detected.

## Data Availability

The data that support the findings of this study are available from the corresponding author upon reasonable request.

## References

[1] Ishihara K., Ueda T., Nakabayashi N. (1990). Preparation of phospholipid polymers and their properties as polymer hydrogel membranes. Polym. J..

[2] Iwasaki Y., Ishihara K. (2012). Cell membrane-inspired phospholipid polymers for developing medical devices with excellent biointerfaces. Sci. Technol. Adv. Mater..

[3] Ueda T., Oshida H., Kurita K., Ishihara K., Nakabayashi N. (1992). Preparation of 2-methacryloyloxyethyl phosphorylcholine copolymers with alkyl methacrylates and their blood compatibility. Polym. J..

[4] Ishihara K., Iwasaki Y. (1998). Reduced protein adsorption on novel phospholipid polymers. J. Biomater. Appl..

[5] Goda T., Ishihara K., Miyahara Y. (2015). Critical update on 2-methacryloyloxyethyl phosphorylcholine (MPC) polymer science. J. Appl. Polym. Sci..

[6] Moro T., Takatori Y., Ishihara K., Konno T., Takigawa Y., Matsushita T., Chung U.I.L., Nakamura K., Kawaguchi H. (2004). Surface grafting of artificial joints with a biocompatible polymer for preventing periprosthetic osteolysis. Nat. Mater..

[7] Ho S.P., Nakabayashi N., Iwasaki Y., Boland T., LeBerge M. (2003). Frictional properties of poly(MPC-co-BMA) phospholipid polymer for catheter applications. Biomaterials.

[8] Lewis A.L., Hughes P.D., Kirkwood L.C., Leppard S.W., Redman R.P., Tolhurst L.A., Stratford P.W. (2000). Synthesis and characterization of phosphorylcholine-based polymers useful for coating blood filtration devices. Biomaterials.

[9] Clarke S., Davies M.C., Roberts C.J., Tendler S.J.B., Williams P.M., O’Byrne V., Lewis A.L., Russell J. (2000). Surface mobility of 2-methacryloyloxyethyl phosphorylcholine-co-lauryl methacrylate polymers. Langmuir.

[10] Lewis A.L., Tolhurst L.A., Stratford P.W. (2002). Analysis of a phosphorylcholine-based polymer coating on a coronary stent pre- and post-implantation. Biomaterials.

[11] Katayama R., Ikeda M., Shiraishi K., Matsumoto A., Kojima C. (2019). Formation of hydrophobic domains on the poly(MPC-co-dodecyl methacrylate)-coated surface recognized by macrophage-like cells. Langmuir.

[12] Katayama R., Tanaka N., Takagi Y., Shiraishi K., Tanaka Y., Matsumoto A., Kojima C. (2020). Characterization of hydration process of phospholipid-mimetic polymers using the air injection-meditated liquid exclusion methods. Langmuir.

[13] Kojima C., Katayama R., Nguyen T.L., Oki Y., Tsujimoto A., Yusa S., Shiraishi K., Matsumoto A. (2020). Different antifouling effects of random and block copolymers comprising 2-methacryloyloxyethyl phosphorylcholine and dodecyl methacrylate. Eur. Polym. J..

[14] Ohshio M., Ishihara K., Yusa S.-I. (2019). Self-association behavior of cell membrane-inspired amphiphilic random copolymers in water. Polymers.

[15] Mu M., Konno T., Inoue Y., Ishihara K. (2017). Solubilization of poorly water-soluble compounds using amphiphilic phospholipid polymers with different molecular architectures. Colloids. Surf. B..

[16] Yusa S.-I., Fukuda K., Yamamoto T., Ishihara K., Morishima Y. (2005). Synthesis of well-defined amphiphilic block copolymers having phospholipid polymer sequences as a novel biocompatible polymer micelle reagent. Biomacromolecules.

[17] Jin Q., Chen Y., Wang Y., Ji J. (2014). Zwitterionic drug nanocarriers: A biomimetic strategy for drug delivery. Colloids Surf. B.

[18] Raval N., Maheshwari R., Shukla H., Kalia K., Torchilin V.P., Tekade R.K. (2021). Multifunctional polymeric micellar nanomedicine in the diagnosis and treatment of cancer. Mater. Sci. Eng. C.

[19] Ghosh B., Biswas S. (2021). Polymeric micelles in cancer therapy: State of the art. J Control. Release.

[20] Lin W., He Y., Zhang J., Wang L., Wang Z., Ji F., Chen S. (2014). Highly hemocompatible zwitterionic micelles stabilized by reversible cross-linkage for anti-cancer drug delivery. Colloids Surf. B.

[21] Cao J., Xie X., Lu A., He B., Chen Y., Gu Z., Luo X. (2014). Cellular internalization of doxorubicin loaded star-shaped micelles with hydrophilic zwitterionic sulfobetaine segments. Biomaterials.

[22] Sill K.N., Sullivan B., Carie A., Semple J.E. (2017). Synthesis and characterization of micelle-forming PEG-poly(amino acid) copolymers with iron-hydroxamate cross-linkable blocks for encapsulation and release of hydrophobic drugs. Biomacromolecules.

[23] Crown J., O’Leary M. (2000). The taxanes: An update. Lancet.

[24] Weiss R.B., Donehower R.C., Wiernik P.H., Ohnuma T., Gralla R.J., Trump D.L., Baker J.R., Van Echo D.A., Von Hoff D.D., Leyland-Jones B. (1990). Hypersensitivity reactions from taxol. J. Clin. Oncol..

[25] Wang F., Porter M., Konstantopoulos A., Zhang P., Cui H. (2017). Preclinical development of drug delivery systems for paclitaxel-based cancer chemotherapy. J Control. Release.

[26] Konno T., Watanabe J., Ishihara K. (2003). Enhanced solubility of paclitaxel using water-soluble and biocompatible 2-methacryloyloxyethyl phosphorylcholine polymers. J. Biomed. Mater. Res. A.

[27] Wada M., Jinno H., Ueda M., Ikeda T., Kitajima M., Konno T., Watanabe J., Ishihara K. (2007). Efficacy of an MPC-BMA co-polymer as a nanotransporter for paclitaxel. Anticancer Res..

[28] Kojima C., Suehiro T., Watanabe K., Ogawa M., Fukuhara A., Nishisaka E., Harada A., Kono K., Inui T., Magata Y. (2013). Doxorubicin-conjugated dendrimer/collagen hybrid gels for metastasis-associated drug delivery systems. Acta Biomater..

